# Clinical relevance of novel Otarine herpesvirus-3 in California sea lions (*Zalophus californianus*): lymphoma, esophageal ulcers, and strandings

**DOI:** 10.1186/1297-9716-43-85

**Published:** 2012-12-12

**Authors:** Stephanie Venn-Watson, Celeste Benham, Frances M Gulland, Cynthia R Smith, Judy St Leger, Pam Yochem, Hendrik Nollens, Uriel Blas-Machado, Jeremiah Saliki, Katie Colegrove, James FX Wellehan, Rebecca Rivera

**Affiliations:** 1 National Marine Mammal Foundation, 2240 Shelter Island Drive, San Diego, California, 92106, USA; 2 The Marine Mammal Center, 2000 Bunker Road, Fort Cronkhite, Sausalito, San Francisco, California, 94965, USA; 3 SeaWorld Parks and Entertainment, SeaWorld San Diego, 500 SeaWorld Drive, San Diego, California, 92109, USA; 4 Hubbs-Seaworld Research Institute, 2595 Ingraham Street, San Diego, California, 92109, USA; 5 Athens Veterinary Diagnostic Laboratory College of Veterinary Medicine, University of Georgia, 501 DW Brooks Dr. Athens, Athens, Georgia, 30602, USA; 6 Zoological Pathology Program, University of Illinois, LUMC Bldg 101 Rm 0745, 2160 S First Avenue, Maywood, Illinois, 60153, USA; 7 College of Veterinary Medicine, University of Florida, 1945 SW 16th Avenue, Gainesville, Florida, 32608, USA

## Abstract

Herpesviruses have been recognized in marine mammals, but their clinical relevance is not always easy to assess. A novel otarine herpesvirus-3 (OtHV3) was detected in a geriatric California sea lion (*Zalophus californianus*), and using a newly developed quantitative PCR assay paired with histology, OtHV3 was associated with esophageal ulcers and B cell lymphoblastic lymphoma in this animal. The prevalence and quantities of OtHV3 were then determined among buffy coats from 87 stranded and managed collection sea lions. Stranded sea lions had a higher prevalence of OtHV3 compared to managed collection sea lions (34.9% versus 12.5%; *p* = 0.04), and among the stranded sea lions, yearlings were most likely to be positive. Future epidemiological studies comparing the presence and viral loads of OtHV3 among a larger population of California sea lions with and without lymphoid neoplasia or esophageal ulcers would help elucidate the relevance of OtHV3-associated pathologies to these groups.

## Introduction

Herpesviruses and herpesvirus antibodies have been identified in marine mammals globally [1-3]. To date, marine mammal herpesviruses have included alphaherpesviruses and gammaherpesviruses [4]. Herpesviruses have been associated with disease outbreaks involving interstitial pneumonia and adrenal necrosis in harbor seals (*Phoca vituliana*) [1,5]; dermatitis in a beluga whale (*Delphinapterus leucas*) [6]; encephalitis in a harbor porpoise (*Phocoena phocoena*) [7]; and fatal disseminated infections, skin lesions, and genital lesions in bottlenose dolphins (*Tursiops truncatus*) [8,9].

*Otarine herpesvirus-1* (OtHV1), a gammaherpesvirus, was identified among California sea lions (*Zalophus californianus*) and has been associated with urogenital carcinoma [10]. OtHV2 was subsequently discovered from an eye swab in a California sea lion; the clinical significance of OtHV2 was unknown [5]. During 2008, a 24-year old geriatric male California sea lion with ulcerative esophagitis died from B cell lymphoblastic lymphoma. A novel herpesvirus, OtHV3, was identified in multiple tissues from this animal. Lymphomas in humans, including B cell lymphomas and leukemias, have been associated with herpesvirus infections [11,12]. While laboratory animal studies have demonstrated that herpesvirus infections can lead to lymphoblastic lymphoma, identifying naturally occurring animal models for herpesvirus infection-associated lymphomas and leukemias may provide further insight into the ecology of this disease [13-15].

To better understand the clinical relevance of OtHV3 infection in California sea lions, blood samples collected from wild stranded and healthy managed collection groups were tested for OtHV3 using both PCR and a newly developed quantitative PCR assay. Prevalence of OtHV3 infections and viral loads were compared among wild and managed collection groups, with the case sea lion, and among wild sea lions by sex, age class, cause of stranding, re-stranding status, the presence of abscesses or pneumonia, and body condition.

## Materials and methods

### Examination of Animal A

The initially identified case animal, heretofore called “Animal A”, was a 24-year old California sea lion that was necropsied by the veterinary staff within 2 h of death using a standardized protocol at the Navy Marine Mammal Program. Gross observations were made on the following tissues: adrenal gland, brain, cavities (oral, peritoneal, and thoracic), esophagus, eye, genital, heart, intestines, kidney, lung, liver, lymph nodes, musculoskeletal, oral cavity, pericardium, skin, spleen, stomach, thyroid, trachea, and urinary bladder. Routine histological evaluations of these tissues were conducted by a reference pathology laboratory using hematoxylin and eosin stained slides made from paraffin-embedded blocks. Additional tissue sets were collected and archived in cryovials at −80°C.

Given the identification of lymphoblastic lymphoma in multiple tissues throughout Animal A, immunochemistry staining for the B cell marker CD20 (rabbit polyclonal antibody; 1:800; Thermo Fisher Scientific, Kalamazoo, MI, USA) and T cell marker CD3 (rabbit polyclonal antibody; 1:150; Biocare Medical, Concord, CA, USA) were conducted on esophageal tissue cut from paraffin-embedded blocks. Positive staining was visualized using diaminobenzidine (DAB) chromogen (Biogenes, San Ramon, CA, USA) Sections of sea lion and dog lymph node were used as positive controls. Negative controls were sections of esophagus incubated with omission of the primary antibody. The B-cell marker CD79a does not cross react with sea lion lymphocytes, Negative stain electron microscopy (NEM) was conducted on liver, kidney, lymph node, and esophageal tissue. Transmission electron microscopy was used on thin sections of esophageal tissue.

### Sample collection, processing and DNA extractions

In addition to tissues collected from the case sea lion, 11 tissues, including kidney, liver, lymph node, skin, spleen, and thyroid, were tested for OtHV3 using PCR in tissues from 10 sea lions from five groups (Navy Marine Mammal Program, SeaWorld Florida, and SeaWorld San Diego, The Marine Mammal Center, and Theater of the Sea). These tissues were provided by the facilities in response to the authors’ request to test other sea lion tissues for OtHV3, and full health histories were not provided. One of the sea lions from The Marine Mammal Center did have a gastrointestinal T-cell lymphoma, and tumor tissue was tested for OtHV3, along with liver, esophagus, cervix, vagina, and urinary bladder. Buffy coats were collected from 87 California sea lions at a rehabilitation center for stranded marine mammals (The Marine Mammal Center, Sausalito, CA, USA; *n* = 63) and a managed collection of sea lions (Navy Marine Mammal Program, San Diego, CA, USA; *n* = 24). Buffy coat and tissue samples were stored without media at −80°C. Samples were later thawed on ice and DNA was extracted (DNeasy Blood and Tissue Kit, Qiagen, Valencia, CA, USA) following the manufacturer’s instructions. DNA extractions were stored at −20°C until PCR analysis.

### Sequence and phylogenetic analyses

Nested PCR amplification of a partial sequence of the herpesvirus DNA-dependent DNA polymerase gene was performed using methods described elsewhere [16]. To obtain additional sequence, the original first round product was used and the second round was altered to use primers DFA and IYG [16]. The PCR products were resolved in 1% agarose gels, excised, and purified (QIAquick gel extraction kit, Qiagen, Valencia, CA, USA). Sanger sequencing was performed directly (Big-Dye Terminator Kit, Applied Biosystems, Foster City, CA, USA) and analyzed on ABI 3130 automated DNA sequencers. Products were sequenced twice in each direction.

Predicted homologous 153–167 amino acid sequences of DNA-dependent-DNA polymerase were aligned using MAFFT [17]. Partial homologous amino acid sequences for which full-length sequence was not available were included, with ambiguities added for unknown amino acids, for Otariid HV2 (55 amino acids), Phocid herpesvirus 5 (150 amino acids), Delphinid HV9 (156 amino acids), and Trichechid HV1 (149 amino acids). Additionally, Iguanid herpesvirus 2 (GenBank accession no. AY236869) was designated as the outgroup due to its early divergence from other herpesviruses [18]. Bayesian analyses of amino acid alignments were performed using MrBayes 3.1.2 [19] on the CIPRES server [20], with gamma distributed rate variation and a proportion of invariant sites, and mixed amino acid substitution models. The first 25% of 1 000 000 iterations were discarded as a burn in. Maximum likelihood (ML) analyses of each alignment were performed using RAxML on the CIPRES server [21], with gamma distributed rate variation and a proportion of invariant sites. The amino acid substitution model with the highest posterior probability in the Bayesian analysis was selected. Bootstrap analysis was used to test the strength of the tree topology [22]. Numbers of bootstrap replicates were determined using the stopping criteria by Pattengale et al. [23].

### OtHV3 quantitative PCR (qPCR) survey

An OtHV3 PCR amplicon from the adrenal gland of the case sea lions, amplified as above, was used as the template to prepare the qPCR standard curve. The OtHV3 cDNA fragment was cloned using the Topo TA Cloning® Kit for Sequencing (Invitrogen, Carlsbad, CA, USA). The plasmid DNA was then PCR amplified using M13 forward and reverse primers and sequenced for confirmation. The amplicon from the cloned OtHV3 fragment was used as the OtHV3 qPCR standard. This standard was quantified by both comparison to a low mass ladder standard (Invitrogen, Carlsbad, CA, USA) on gel electrophoresis as well as by spectrophotometry (NanoDrop 8000, Thermo Scientific, Wilmington, DE, USA). The standard was diluted in 10-fold serial dilutions ranging from 10^8^ to 10 copies using nuclease-free water, and subsequently run with each assay.

Quantitative PCR was performed using forward primer OtHV3qPCRF (5’-GGACGGAGGATGCTAGACAA-3’), reverse primer OtHV3qPCRR (5’-CCGGAAAAGCGTTCTAGGTC-3’) (Sigma-Aldrich, St. Louis, MI, USA), and OtHV3probe (6FAM-TAGAAAAGCTTTCCCTGGGG) (Applied Biosystems, Carlsbad, CA, USA) targeting the DNA-dependent DNA polymerase gene (amplicon length 74 bp). All samples were run in duplicate and a mean Ct value was calculated. A Eukaryotic 18S rRNA Endogenous Control kit (VIC/MGB Probe, Applied Biosystems, Foster City, CA, USA) was used to validate the presence of amplifiable DNA in each sample in a separate well. Nuclease-free water was used as a negative control. DNA extracted from a buffy coat known to contain the virus was used as a positive control and was run on each plate. Each 20 μL reaction was 0.9 μM for each primer and 0.25 μM for the probe, and contained 4 μL of extracted DNA and 10 μL of a commercial universal qPCR mix (TaqMan® Fast Universal PCR Master Mix 2X, Applied Biosystems, Life Technologies, Grand Island, NY, USA) a rehabilitation center for stranded marine mammals, including California sea lions. Buffy coats were archived on animals from both facilities. Additionally, standardized medical history information was collected on TMMC sea lions, including sex, age class, re-stranding status, body condition, and cause of stranding such as trauma, abscesses, domoic acid toxicosis, or pneumonia. Ages were classified into pup, yearling, juvenile, subadult, and adult. Age, sex and cause of stranding were determined as described in Grieg et al. [24].

SAS Version 9.2 (SAS Inc., Cary, North Carolina) was used for data analysis. OtHV3-positive and negative sea lions were compared using Chi square tests among the two study groups; stranded sea by sex, age class, re-stranding status and body condition; and stranded sea lions with or without trauma, abscesses, domoic acid toxicosis, and pneumonia. Additionally, stepwise regression was used with a model that included dummy variables of all potential predictors of OtHV3 status among stranded sea lions. OtHV3 loads among positive samples were compared between the two groups using a nonparametric Kruskal-Wallis test. This test was also used to compare differences in OtHV3 loads among stranded sea lions by sex, age class, re-stranding status and body condition; and among those with or without trauma, abscesses, domoic acid toxicosis, and pneumonia. Significance was defined as a *P* value less than or equal to 0.05.

## Results

### Animal A

The 24 year old male California sea lion, Animal A, from which OtHV3 was originally detected had severe and acute lymphocytic leukocytosis (12 660 lymphocytes/μL, reference range 211 to 2 775 cells/μL; and 42 200 white blood cells/μL, reference range 2 432–7 610 cells/μL). The sea lion died within a day of detecting this blood abnormality and had normal total lymphocyte and white blood cell counts five days before death (356 and 8 900 cells/μL, respectively). Blood lymphocytes during the lymphocytosis event were characterized as large, monomorphic, and immature; all of these changes were suggestive of lymphoid neoplasia. Unfortunately, the lymphocytes were not evaluated by a clinical pathologist the reference laboratory, and no slide images were preserved.

**Figure 1:**
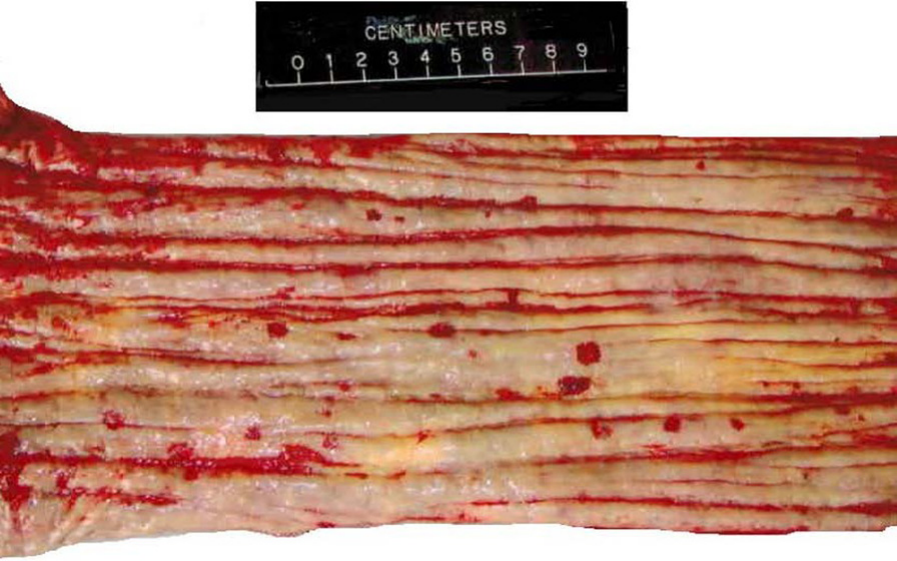
**Multifocal ulcerations throughout the esophageal mucosa of a 24-year old male California sea lion (*Zalophus californianus*). **On gross necropsy evaluation, this sea lion had numerous erosive and ulcerative lesions in the distal half to third of the esophagus. At the esophageal-stomach junction there were large (approximately 1 by 2 cm) erosions. The mucosal lining of the stomach had multiple ulcerative lesions that varied in color from pink to black

**Figure 2:**
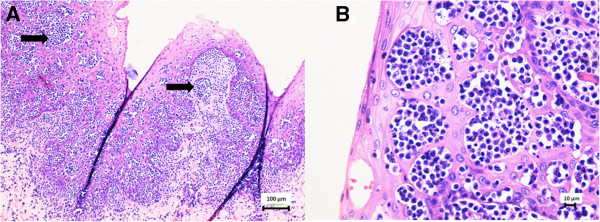
**Hematoxylin and eosin stain of proximal esophagus from a 24-year old male California sea lion (*Zalophus californianus*) with lymphoblastic lymphoma.** Multicentric lymphoblastic lymphoma involving the esophagus was diagnosed in a geriatric sea lion by routine histopathology. Similar findings were identified in the adrenal gland, urinary bladder, colon, heart, kidneys, liver, lymph nodes, pancreas, spleen, stomach, tonsil, thyroid, trachea, tongue, and ureter. Aggregates of neoplastic cells were scattered within affected tissues and often clustered at mucosal and submucosal junctions or within mucosal epithelium. **A**. Clusters of neoplastic cells (arrow) are present in the mucosa, submucosa, and in submucosal blood vessels. **B**. High power (400X) magnification of the moderate to large neoplastic round cells with large round to indented nuclei and coarsely stippled chromatin

On gross necropsy evaluation, this animal had numerous erosive and ulcerative lesions in the distal half to third of the esophagus (Figure 1). At the esophageal-stomach junction there were large (approximately 1 by 2 cm) erosions. The mucosal lining of the stomach had multiple ulcerative lesions that varied in color from pink to black. Multicentric lymphoblastic lymphoma involving the adrenal gland, urinary bladder, colon, esophagus, heart, kidneys, liver, lymph nodes, pancreas, spleen, stomach, tonsil, thyroid, trachea, tongue, and ureter was diagnosed by routine histopathology. The moderate to large neoplastic cells round cells had large round to indented nuclei with coarsely stippled chromatin. Aggregates of neoplastic cells were scattered within affected tissues and often clustered at mucosal and submucosal junctions or within mucosal epithelium (Figure 2). Many blood vessels were dilated and contained neoplastic cells.

**Figure 3:**
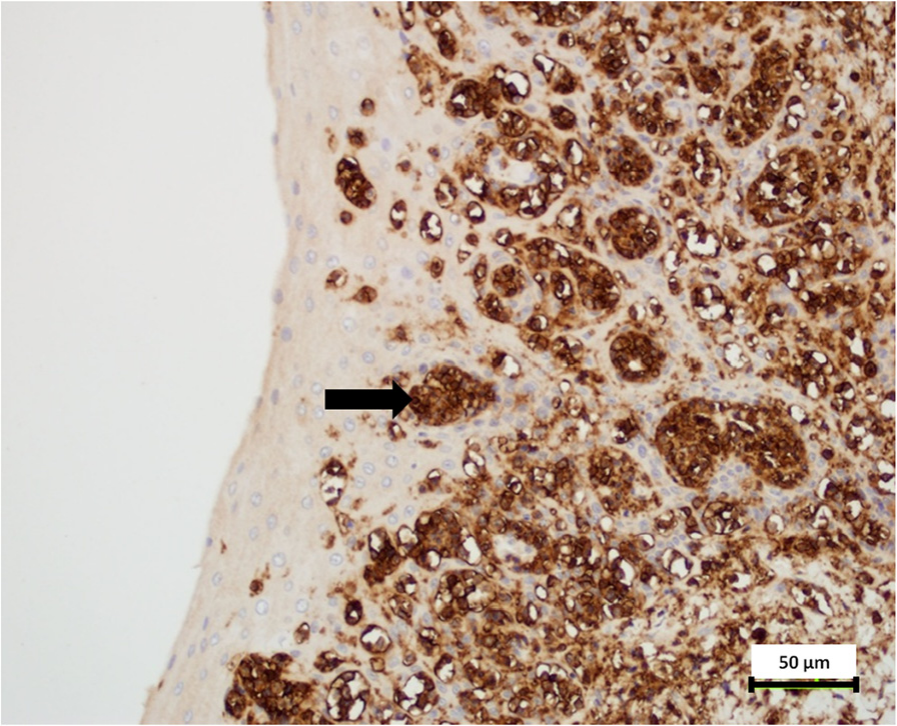
**Esophagus from a 24-year old male California sea lion (*Zalophus californianus*) with B cell lymphoblastic lymphoma.** Neoplastic cells diffusely express the B cell marker CD20. DAB chromogen. 400X magnification. The cells were negative for the T-cell marker CD3. Similar aggregates of neoplastic cells were identified in multiple tissues, including the adrenal gland, urinary bladder, colon, heart, kidneys, liver, lymph nodes, pancreas, spleen, stomach, tonsil, thyroid, trachea, tongue, and ureter

**Figure 4:**
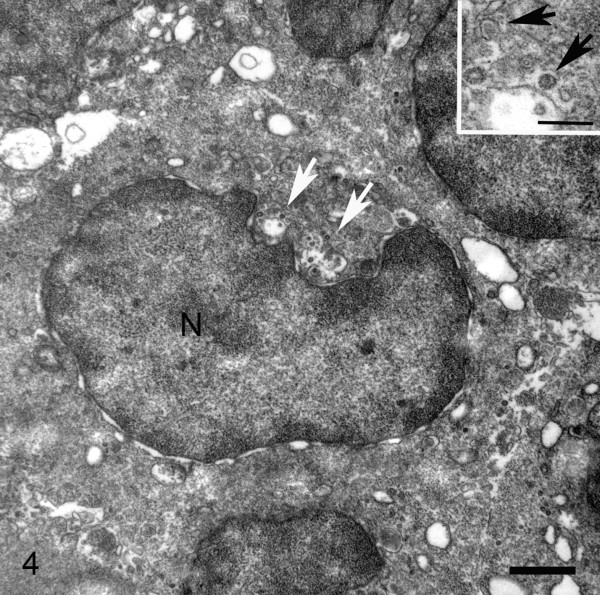
**Esophagus, 24-year old male Sea Lion (*Zalophus californianus*).** Transmission electron ultrastructural image of a B-lymphocyte within the esophageal mucosa. Adjacent to the nucleus (N) are two clusters of membrane-associated, recently formed viral particles budding off the nuclear membrane (white arrows). Inset shows higher magnification of viral particles, with a characteristic electrondense core and capsid (black arrows). Individual particles are 100–150 nm in diameter. Figure 4 bar = 1 μm; inset bar = 250 nm

Neoplastic cells were diffusely positive for CD20, indicating that the cells were of B cell origin; the cells were negative for the T-cell marker CD3 (Figure 3). Among liver, kidney, lymph node, and esophagus, only esophageal tissue had suspicious structures suggestive of, but not distinctly characterized as viral particles using NEM. TEM, however, successfully identified 100 to 150 nm diameter particles in the cytoplasm of a lymphocyte within the esophageal mucosa (Figure 4); particles had a distinct core and capsid and were consistent with herpesvirus (Figure 4).

### Sequence and phylogenetic analyses

**Figure 5:**
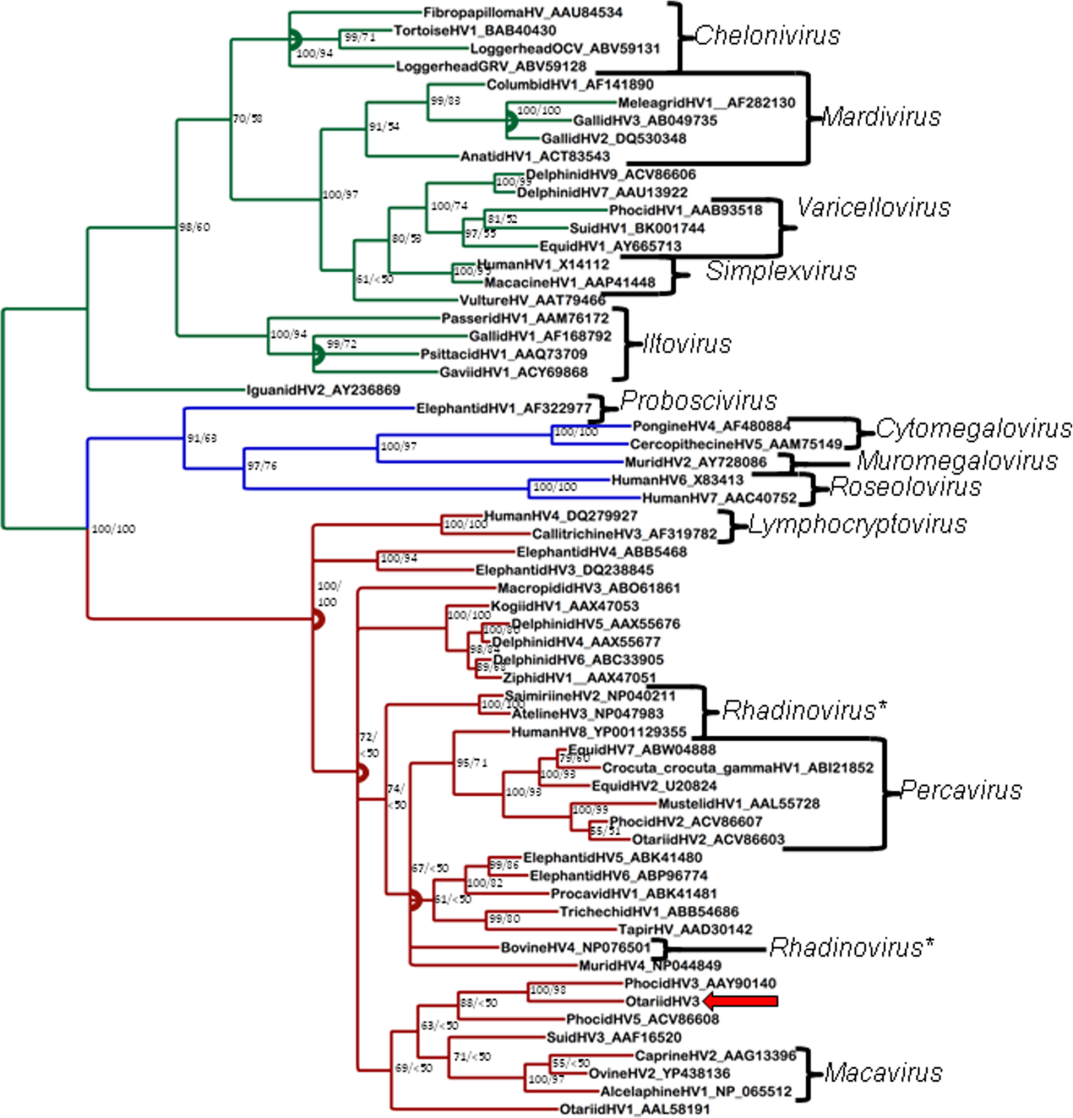
**Bayesian phylogenetic tree of predicted amino acid sequences of herpesviral DNA-dependent-DNA polymerase sequences based on MAFFT alignment.** Multifurcations are marked with arcs. Bayesian posterior probabilities of clusters as percentages are in bold, and ML bootstrap values for clusters based on 450 re-samplings are given to the right. Iguanid herpesvirus 2 was used as an outgroup. OtHV3 is indicated with arrows. The subfamily Alphaherpesvirinae is marked in green Betaherpesvirinae is in blue, and Gammaherpesvirinae is in red. Brackets demarcate genera. Accession numbers of sequences retrieved from GenBank are given after the name

Nested PCR amplification using DFA/IYG resulted in a product of 472 base pairs after primers were edited out. Sequence was submitted to GenBank under accession number JX080682. The Bayesian tree is shown (Figure 5). Bayesian phylogenetic analysis found that the WAG model of amino acid substitution was most probable with a posterior probability of 1.000, and this was used for ML analysis [25]. Stopping criteria for ML bootstrapping were reached after 450 subsets. Bootstrap values as percentages from ML analysis are shown on the Bayesian tree (Figure 5). The analyses found strong support for clustering with PhocidHV3, a gammaherpesvirus, which was identified in Hawaiian monk seals (*Monachus schauinslandi*) [26]. There was weak Bayesian support and no significant ML support for these viruses clustering with the genus *Macavirus*.

### OtHV3 quantitative PCR (qPCR) survey

**Figure 6:**
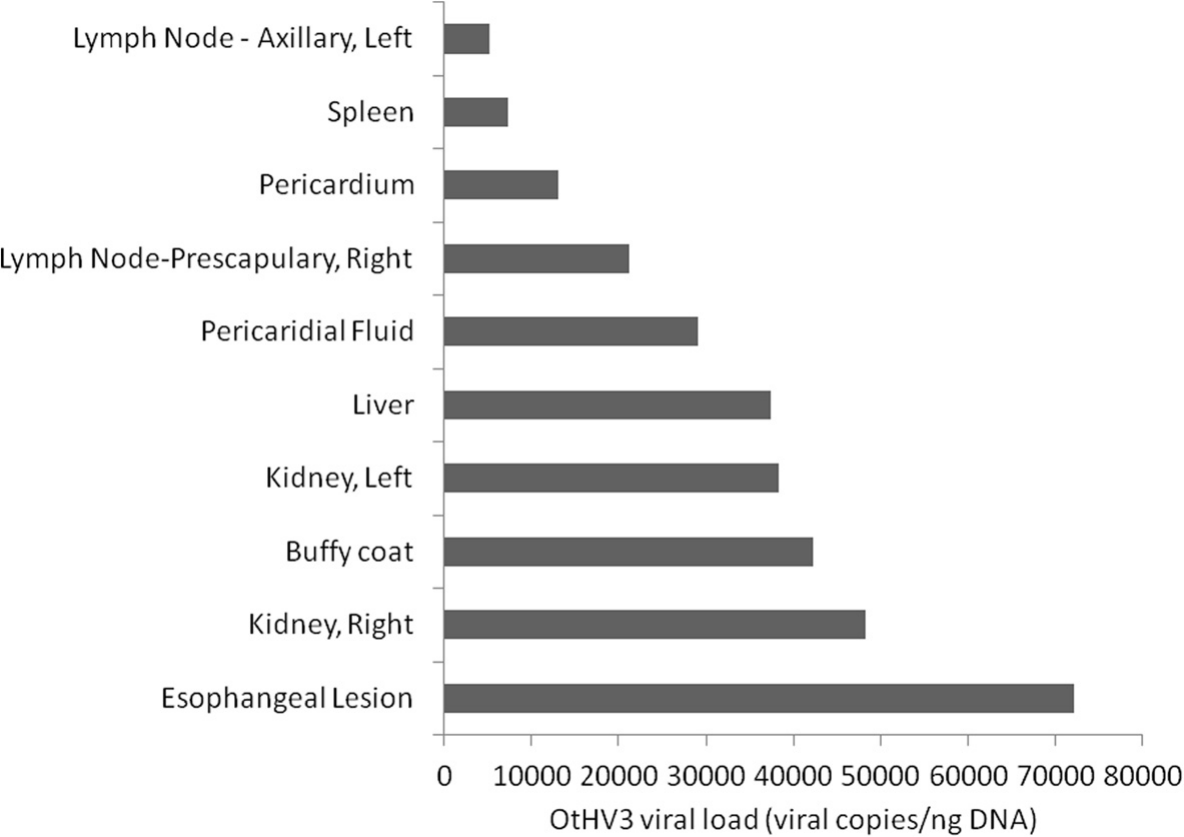
**OtHV-3 copies per ng DNA detected in tissues collected postmortem in a 24-year old male California sea lion (*Zalophus californianus*) with lymphoblastic lymphoma.** The esophagus, kidney, liver, lymph node, pericardial fluid, pericardium, and spleen were qPCR-positive for high copy numbers of OtHV3 (median = 29 009, range 5 205–72 164 viral copies/ng DNA). The esophagus had the highest OtHV3 viral load. A buffy coat collected from Animal A eight months prior to death was qPCR negative for OtHV3. Of 10 other sea lions that had postmortem tissue tested, five (50%) had at least one tissue with detectable OtHV3. Copy numbers among those OtHV3 positive sea lions, however, were much lower than those of the sea lion with lymphoblastic lymphoma (ranges = 0.01 to 0.22 versus 5 205 to 72 164 viral copies/ng DNA)

The qPCR assay worked well, with a calculated efficiency ≥ 96% and R^2^ ≥ 99%, and reliably detected 10 copies. For Animal A, the esophagus, kidney, liver, lymph node, pericardial fluid, pericardium, and spleen were qPCR-positive for high copy numbers of OtHV3 (median = 29 009, range 5 205–72 164 viral copies/ng DNA) (Figure 6). The esophagus had the highest OtHV3 viral load. A buffy coat collected from Animal A eight months prior to death was qPCR negative for OtHV3.

Of 10 sea lions (excluding Animal A) that had postmortem tissue tested, five (50%) had at least one tissue with detectable OtHV3. Tissues that were OtHV3 positive were thyroid gland (*n* = 1), liver (*n* = 1), spleen (*n* = 2), skin (*n* = 1), and a lymph node with T-cell lymphoma (*n* = 1). Copy numbers among the OtHV3 positive sea lions, however, were much lower than those of the case sea lion (ranges = 0.01 to 0.22 versus 5 205 to 72 164 viral copies/ng DNA).

Of buffy coats collected from 87 California sea lions from August 2006 to July 2010, the qPCR successfully detected OtHV3 in 25 (28.7%) animals. TMMC stranded sea lions were more likely to be OtHV3-positive compared to the Navy's healthy sea lions (22/63, 34.9% versus 3/24, 12.5%; *P* = 0.04). When removing Animal A, there were no significant differences in OtHV3 viral loads in positive animals from the Navy sea lions compared to TMMC stranded sea lions (0.19 ± 0.14 and 0.14 ± 0.18 viral copies/ng DNA, respectively *P* > 0.05).

### Clinical relevance of OtHV3-positive buffy coats in wild sea lions

Stranded sea lions that were OtHV3-positive were 5.1 times more likely to be yearlings (71.4%) and 3.9 times more likely to have pneumonia (59.1%) compared to OtHV3-negative sea lions (35.3% yearlings; 26.8% pneumonia; *P* = 0.01). OtHV3 positive sea lions were less likely to have stranded due to domoic acid toxicosis compared to OtHV-3 negative sea lions (9.1% and 36.6%, respectively; *P* = 0.04). When using multivariate stepwise regression, however, only yearling status remained a significant predictor of OtHV3 positive status (*P* = 0.009). There were no differences in the prevalence of sea lions that were female, malnourished, trauma, abscess, or re-stranded sea lions when comparing OtHV3 positive and negative animals.

## Discussion

This study identified a novel herpesvirus, OtHV3, in the buffy coats of both wild stranded and healthy California sea lions from a managed collection. While OtHV3 was present in both groups, viral loads identified in the case sea lion that died from B cell lymphoma were magnitudes higher compared to the rest of the study population; the median and range of viral loads in Animal A was 29 009 and 5 205–72 164, while tissue loads from other sea lions ranged from 0.01 to 0.22 viral copies/ng DNA. Interestingly, OtHV3 loads were high in the same organs that were described as having lymphoblastic lymphoma upon histopathology.

Lymphomas have been associated with herpesvirus infection in humans, including HHV4 with Burkitt’s lymphoma and other B-cell lymphoproliferative disorders [12]. While research on viral-associated lymphoproliferative disorders in humans is often limited to epidemiological associations between herpesvirus infections and disease, research with animal models has demonstrated cause and effect. Experimental inoculation of owl monkeys (*Aotus* sp.) with Saimiriine herpesvirus 2 successfully induced acute lymphocytic leukemia; this outcome was demonstrated using intravenous, subcutaneous, and intradermal inoculations [14]. Similar studies have been reported with marmosets and rabbits [13,15]. Multiple types of human herpesviruses have been associated with patients with B cell acute lymphoblastic leukemia, especially HHV4 and HHV5 [11]. While the clinical pathology of Animal A was suggestive of acute lymphoblastic leukemia (ALL), the acute versus chronic state of neoplasia in this animal was not examined.

In addition to lymphoma, Animal A had numerous esophageal ulcerative lesions in the distal half to third of the esophagus. Interestingly, the highest viral load found in the case animal was from one of these esophageal ulcers. In humans, the esophagus is the most common nongenital viscera affected by herpesvirus infections, with one study demonstrating that 41 of 50 people with herpes lesions in the esophagus postmortem had no other body part affected [27]. Additionally, neoplastic lymphocytes were clustered in the esophageal mucosa and submucosa which could have caused the ulceration and accounted for the high viral load.

In this study, wild stranded sea lions were more likely to be OtHV3-positive by PCR compared to healthy sea lions from a managed collection. Among the stranded sea lions, those that were yearlings were at the highest risk of OtHV3 infection compared to other age classes. While most of the yearlings in the study had pneumonia, in California sea lions, verminous pneumonia is extremely common in yearling animals due to the high prevalence of the lungworm *Parafilaroides decorus*; thus the role of a herpesvirus in these cases is hard to determine [28].

Human herpesviruses, including HHV1, HHV2, HHV4, HHV5, HHV6, and HHV7 have been associated with pneumonia. The prevalence of herpesvirus-associated pneumonia is higher among immunocompromised adults compared to those with competent immune systems, and these pneumonias can be due to endogenous reactivation of latent viral infections or introduction of a new virus [29]. The characterization of pneumonia may be an indicator of herpes infection source; in humans, focal lung infections likely originate from the alveoli, while diffuse interstitial pneumonia may be more likely to be from blood-borne dissemination [30].

Herpesviruses have been associated with pneumonia, primarily interstitial pneumonia, in terrestrial mammals, including cattle, donkeys, horses, and mice [31-34]. Bovine shipping fever pneumonia is due to co-infections by *Mannheimia haemolytica* or *Pasteurella multocida* with a viral infection, including bovine herpesvirus 1 [34]. Similar bacterial-herpesvirus co-infections are found in equine, in which equine herpesvirus type 2 is a predisposing factor for *Rhodococcus equi* pneumonia in foals [32]. As such, evaluating the potential for bacterial-viral coinfections among yearling sea lions may be an area for future research.

In the current study, it is also plausible that increased viral levels are resultant from rather than causal to concurrent disease. In other mammals, stress is well documented as a cause of herpesvirus reactivation, and different stress factors have been shown to have different effects on herpesviral reactivation [35-39]. The finding of a greater prevalence of OtHV3 in sea lions stranding for reasons other than domoic acid toxicosis may reflect increased stress. There was however, no significant difference in viral loads when comparing the different groups, indicating that reactivation of a latent infection was not present.

Our phylogenetic analyses were largely in agreement with previous herpesviral phylogenies. All herpesviral genera were strongly supported, with the exception of *Rhadinovirus*. Historically, the Gammaherpesvirinae were divided into two genera, *Lymphocryptovirus* and the more speciose *Rhadinovirus*. More recently, *Macavirus* and *Percavirus* were recognized as distinct clades and split out from *Rhadinovirus*, leaving it as somewhat of a catch-all genus [40]. The analyses found strong support for OtHV3 clustering with PhocidHV3, which was identified in Hawaiian monk seals [26]. Although PhocidHV4, from Northern elephant seals (*Mirounga angustirostris*) [41], was not included in this analysis, it would have certainly fallen in this cluster due to its extremely close relationship to PhocidHV3 (with 98% amino acid identity over this region, as compared to 71% identity between OtHV3 and PhocidHV3). In the Bayesian but not ML analysis, Phocid HV5 (from harbor seals (*Phoca vitulina*) [4]) was also supported as clustering with OtHV3/Phocid HV3, forming a pinniped gammaherpesvirus clade.

It should be noted that the qPCR values are for copies of target DNA detected, which may potentially differ from actual copy numbers and virion present. OtHV3 has not been successfully cultured, and thus, the actual numbers of whole virus in the samples remain unknown. We used dilutions of known copy numbers of OtHV3 DNA as a standard curve. This control is a DNA template and does not reflect loss during extraction. The presence of PCR inhibitors or nucleases may result in falsely low readings. Further, extraction efficiency may differ between tissues and/or other samples, so caution should be used to avoid over-interpretation when comparing different sample types. In this study, a handful of organ tissues from ten sea lions other than Animal A were opportunistically tested for OtHV3 DNA. It is important to note that the qPCR assay was developed for blood, not tissues, and further work is needed to further validate whether or not low levels of OtHV3 were truly present in different organs of different animals.

In conclusion, OtHV3 infected California sea lions may potentially be natural animal models for herpesvirus-associated diseases, including pneumonia and lymphoma. Future studies should further assess potential associations between OtHV3 infections and these diseases in California sea lions.

## Abbreviations

HV: Herpesvirus; HHV: Human herpesvirus; ML: Maximum likelihood; MMP: Navy Marine Mammal Program; NEM: Negative stain electron microscopy; OtHV: Otarine herpesvirus; PCR: Polymerase chain reaction; qPCR: Quantitative polymerase chain reaction; TEM: Transmission electron microscopy; TMMC: The Marine Mammal Center; WAG: Whelan and Goldman.

## Competing interests

The authors declare that they have no competing interests.

## Authors’ contributions

SVW designed, analyzed, and interpreted the epidemiological and case studies and drafted the manuscript. All other authors critically reviewed the manuscript and provided final approval. CB and RR carried out the molecular studies. FMG, PY, and JSL acquired the wild sea lion samples and health data. CRS acquired the case sea lion samples and health data. JW and HN interpreted the molecular data. UBM, KC, and JS conducted histopathological interpretations of the case animals’ tissues, including transmission electron microscopy and immunohistochemistry.
